# Mitochondrial Complex I and β-Amyloid Peptide Interplay in Alzheimer’s Disease: A Critical Review of New and Old Little Regarded Findings

**DOI:** 10.3390/ijms242115951

**Published:** 2023-11-03

**Authors:** Anna Atlante, Daniela Valenti

**Affiliations:** Institute of Biomembranes, Bioenergetics and Molecular Biotechnologies (IBIOM), National Research Council (CNR), Via G. Amendola 122/O, 70126 Bari, Italy; d.valenti@ibiom.cnr.it

**Keywords:** Alzheimer’s disease, mitochondrial dysfunction, mitochondrial complex I, amyloid-β peptide, reactive oxygen species

## Abstract

Alzheimer’s disease (AD) is the most common neurodegenerative disorder and the main cause of dementia which is characterized by a progressive cognitive decline that severely interferes with daily activities of personal life. At a pathological level, it is characterized by the accumulation of abnormal protein structures in the brain—β-amyloid (Aβ) plaques and Tau tangles—which interfere with communication between neurons and lead to their dysfunction and death. In recent years, research on AD has highlighted the critical involvement of mitochondria—the primary energy suppliers for our cells—in the onset and progression of the disease, since mitochondrial bioenergetic deficits precede the beginning of the disease and mitochondria are very sensitive to Aβ toxicity. On the other hand, if it is true that the accumulation of Aβ in the mitochondria leads to mitochondrial malfunctions, it is otherwise proven that mitochondrial dysfunction, through the generation of reactive oxygen species, causes an increase in Aβ production, by initiating a vicious cycle: there is therefore a bidirectional relationship between Aβ aggregation and mitochondrial dysfunction. Here, we focus on the latest news—but also on neglected evidence from the past—concerning the interplay between dysfunctional mitochondrial complex I, oxidative stress, and Aβ, in order to understand how their interplay is implicated in the pathogenesis of the disease.

## 1. Introduction

**Figure 1:**
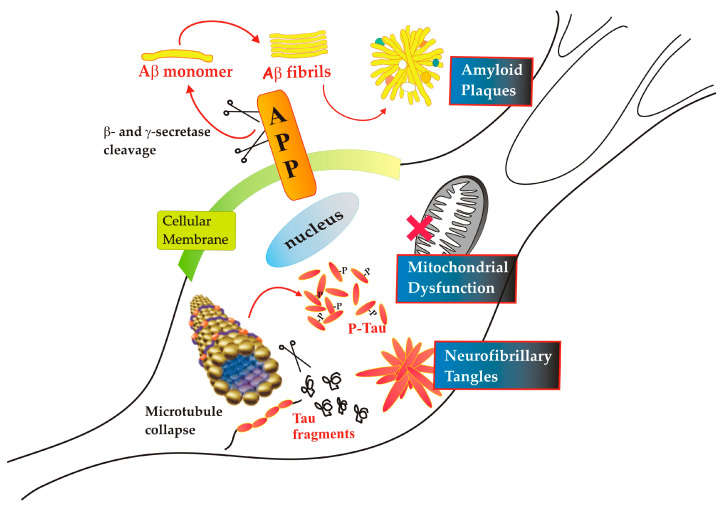
The most distinctive diagnostic features of Alzheimer’s disease: amyloid plaques, i.e., accumulations of β-amyloid (Aβ) protein outside neurons; neurofibrillary tangles of abnormally modified Tau protein, i.e., Phosphorylated-Tau (P-Tau) and/or Tau fragments, inside cells; mitochondrial dysfunctions.

«Ich habe mich verloren»—translated from German «I lost myself»—are the few words that Auguste Deter obsessively repeated when Dr. Alois Alzheimer, who was treating her, asked her to write her name. From a careful post-mortem examination of Auguste’s brain, alterations typical of the disease that afflicting the woman were found, i.e., intra- and extracellular accumulations of protein material. These accumulations interfere with the functioning of individual neurons, inducing cellular suffering and loss of synapses, and subsequently, neuronal death. They are the most distinctive diagnostic features of Alzheimer’s disease (AD), the identity of which remained unknown until the mid-eighties. The so-called amyloid plaques, i.e., accumulations of β-amyloid (Aβ) protein outside neurons, are clumps that form between neurons and damage surrounding cells, while tangles of abnormally modified Tau protein inside cells block communication between nerve cells [1] (Figure 1).

Aβ, a particularly toxic variant prone to aggregation, results from incorrect cleavage of the amyloid precursor protein (APP), a transmembrane protein known to be the precursor of Aβ. The physiological or pathological processing of this polypeptide occurs by α-, β-, and γ-secretases [2]. The concerted action of β- and γ-secretase gives rise to the release of Aβ1-42 (which we will call from now on Aβ), the anomalous peptide made up of 42 amino acids, while α-secretase represents the main player in the physiological metabolic processing of APP [3]. It is the Aβ that aggregates into fibrils that accumulate in the extracellular matrix surrounding neuron cells to form plaques between one cell and another, thus exerting a harmful action on the surrounding healthy cells [3,4]. Although it is not yet entirely clear in which form—monomeric, dimeric, or polymeric—Aβ exerts its toxic action, the scientific community recognizes the excessive production of this peptide as the triggering cause of the complex series of events that lead to neuronal death [5]. Hence, numerous studies have focused on understanding the toxicity of Aβ and its relationship with AD progression [6,7,8,9].

Within the same neurons, neurofibrillary tangles (NFT) have been shown to consist of abnormal forms of the Tau protein [10], originally discovered in 1975 by Weingarten et al. [11] as a protein that co-purified with microtubules and was precisely called Tau due to its ability to induce “tubule formation”. The Tau protein undergoes a series of post-translational modifications and attacks by proteolytic enzymes which end with its partial demolition and formation of protein spirals, called NFT [12,13]. Hyperphosphorylation and degradation of Tau are thought to underlie its tendency to aggregate [12]. The detachment of Tau from the microtubule causes its collapse which, in turn, materializes in the arrest of axonal transport and in the death of the entire neuron [13,14].

The two toxic protein compounds, Aβ and Tau, come into action at different temporal stages of the disease showing different degrees of harmfulness [15,16]. Researchers have identified Aβ accumulation as a marker for the early stage of AD and phosphorylated Tau (pTau) as an indicator for advanced stages of disease [13,17].

At the current state of knowledge, the crucial question to ask is which upstream molecular events and which mechanisms determine the alterations of Aβ and Tau leading to the formation of plaques and/or tangles? A unifying hypothesis on the causes of the disease identifies the triggering event as apoptosis affecting large neuronal populations. In fact, it is known that neurons activate their own endogenous death program by apoptosis often for unknown causes, or due to the lack of trophic factors whose function it is to keep the apoptotic program blocked (see [18]). Consistently, based on this hypothesis, a cause that can activate an apoptosis program in cell cultures or in laboratory animals is the deprivation of electrical stimuli. It has been observed that if, in vitro, the potassium concentrations that are normally used to keep the nerve cells alive are reduced—since the presence of this cation simulates a condition of electrical stimulation—an equal apoptotic process is activated. Furthermore, this event is accompanied by increased production and release of Aβ and extracellular fibril formation which are found in the disease affecting humans [19,20]. When the block of the apoptotic program is missing, the nerve cells eliminate themselves and depending on the functions they perform in the brain, memory, cognitive abilities, and movement are seriously compromised. If the hypothesis of an anomalous activation of apoptotic processes as a trigger of AD is correct, we should hypothesize that this mechanism causes an anomalous processing of APP and Tau [21].

Consequently, this hypothesis raises numerous other questions: what are the mechanisms that would trigger the initial apoptotic process that causes the hypothesized chain reaction? Furthermore, why, fortunately, is this “pathological” process activated only in a small proportion of the elderly, in whom a “physiological” loss of neurons occurs anyway? Other questions still remain.

Today, the scenario of the intense Alzheimer’s research has changed quite a bit because the most accredited hypothesis of the primary pathogenic role played by the accumulation of extracellular plaques in some regions of the brain, crucial for cognitive functions, is not free from weak points: (i) older patients, with a great accumulation of amyloid plaques, do not show severe cognitive deficits, while other patients, despite a minor load of amyloid aggregates, manifest more serious symptoms of dementia; (ii) many therapies developed to treat the disease by targeting the Aβ protein have had very limited success. Then, it became clear that the path to follow to treat the pathology does not consist in targeting the different forms of Aβ, but in exploring other paths and, at the same time, in re-evaluating/revisiting the role of Aβ in the pathology, knowing with certainty that Aβ plays a critical function, but perhaps not the one imagined up to this point by scientists. Therefore, at the moment, Tau and Aβ are in the back space of the stage, leaving new actors on the scene and with a new, rather elaborate script. The growing idea is that the neuropathogenesis of AD is not causally related to the accumulation of Aβ alone, followed by the appearance of modified Tau, but rather that AD is a multifactorial disease [22,23], where plaques and accumulations of Aβ are even late manifestations.

One of the most promising theories puts mitochondrial dysfunction at the center of the scene, in the role of the major responsible process for the reduction in the brain’s metabolic energy efficiency associated with the increase in oxidative stress. In animal models that develop typical AD symptoms, mitochondrial abnormalities are observed already in the embryonic stages and in young mice, long before the accumulation of Aβ. It is even believed that malfunctions of the mitochondria can occur 20 years before the symptoms of the disease manifest [24,25,26].

This change of scene should push the scientific community to not only focus on the two toxic proteins as the cause responsible for the onset of AD—in particular on Aβ which, more than a hypothesis, has become almost a dogmatic belief—but to focus research on other aspects, such as targeting mitochondrial function, which we believe may offer new potential therapeutic opportunities. Certainly, it cannot be ruled out that proteinopathy together with mitochondrial dysfunction and oxidative stress—as we will see below—could work in concert, underlying the pathology in the aging brain (Figure 1).

In particular, in this report, we will critically review and particularly focus on studies investigating the interplay between dysfunctional mitochondrial complex I, oxidative stress, and Aβ, in order to obtain an in-depth understanding of the causal or correlative relationship between mitochondrial functional alterations and Aβ toxicity and their relationship with the progression of AD, with exclusive reference to the AD sporadic form.

## 2. Mitochondrial Dysfunction and Oxidative Stress in AD

Mitochondrial dysfunction has been proposed as a key event in the etiology of AD (for refs, see [8,27,28,29]). Exploring this fascinating topic could open new and interesting scenarios for research aimed at investigating the molecular bases of the causal correlation between mitochondrial alterations and the onset of AD, as well as constituting a useful prerequisite to orient clinicians towards early and preventive interventions targeting mitochondria, given that it is believed that the malfunction of the mitochondria can occur 20 years before the person shows symptoms of the disease.

Molecular, cellular, gene expression, and immunochemical studies of post-mortem human AD brains, brain samples from AD transgenic mice, platelets from AD patients, and cell lines expressing mutant APP and/or Aβ-treated cells (for ref, see [30]) have revealed multitarget dysfunction of the mitochondrial electron transfer chain (ETC) [25,28,30]. This can be explained by the functional alterations of one or more complexes of the mitochondrial ETC, responsible for the impairment of oxidative phosphorylation (OXPHOS) machinery and severe impairment of mitochondrial electrochemical potential generation. In addition, in several AD models, the deregulation of calcium flow within the mitochondria was found, thus depressing the activity of enzymes responsible for energy production [31], reactive oxygen species (ROS) overproduction [32], impairment of mitochondrial anterograde transport along the neuronal axon [33], and altered mitophagy [34].

However, it is unclear whether these ETC deficits observed in AD are the result of primary mitochondrial dysfunction, as suggested in the mitochondrial hypothesis of AD [27], or a consequence of Aβ accumulation within the brain of patients suffering from AD. Consistently, evidence obtained from AD animal and cellular models suggest that both amyloid and Tau have a direct effect on ETC function (for ref, see [27]). Confirmation of this comes from the triple transgenic 3xTg-AD mouse model (human APPSWE, TauP301L, and PS1M146V genes) in which abnormalities in mitochondrial function are seen in the embryonic stage and in young mice long before amyloid accumulation (for ref, see [27]). Starting from the assumption that the Krebs cycle (TCA) and the ETC complexes (I, II, III, and IV) represent the mitochondrial metabolic pathways that respectively provide the reduced substrates and generate the proton gradient across the internal membrane of the mitochondria used to produce energy in the form of ATP, several studies have demonstrated a strong decrease (30–40%) in the activity of complex IV (see [35,36]), as well as in mitochondrial isocitrate dehydrogenase, pyruvate dehydrogenase, α-ketoglutarate dehydrogenase, and the ATP synthase complexes [34,37,38,39], severely compromising energy metabolism, as demonstrated by the decrease in glucose utilization in the hippocampus, cortex, and posterior cingulate cortex [24,40], and the shortage in mitochondrial ATP production [41,42]. Interestingly, the activities of all these enzymes are inhibited by Aβ [43] providing a possible link between the amyloid cascade and mitochondrial dysfunction in AD.

However, while several groups failed to find substantial differences in the protein expression pattern of OXPHOS in AD compared to the control, others even found increased expression levels of OXPHOS genes in AD, which could be due to the different brain regions analyzed and/or the extensive heterogeneity of the sporadic AD brain samples used (for refs, see [24]). For example, reduced expression levels of complexes I, II, IV, and V were assayed in the entorhinal cortex (EC), but not in the frontal cortex in AD Braak stages V-VI compared to stages I-II [44], which, as is known, indicate the histopathological staging system, according to which early Braak stages are associated with isolated memory impairment, whereas Braak stages V–VI are incompatible with normal cognition [45]. Furthermore, in triple transgenic 3xTg-AD mice (human APPSWE, TauP301L, and PS1M146V genes), mitochondrial dysfunctions are observed already in the embryonic stage and in young mice, long before Aβ accumulation [27,46]; in this same AD mouse model, most subunits of complexes I and IV are downregulated and complexes III and V are upregulated in mitochondria isolated from 6-month-old mice [47].

As part of the study of the relationship between amyloid plaque deposition and glucose metabolism, multimodal imaging studies, using positron emission tomography (PET)— one of the diagnostic tools available in the management of the disease [48]—with both biomarkers, fluorodeoxyglucose and amyloid, have identified the existence of intricate cellular mechanisms that favor the survival of some neurons even in an environment made toxic by Aβ peptides [49]. The reduction in ROS, together with the elevated activity of pyruvate dehydrogenase kinase 1 and lactate dehydrogenase A, in the Aβ-resistant nervous cell lines [50], suggest the possible activation of anti-apoptotic mechanisms due to the Warburg effect [49]. Abnormalities of intracellular glucose transport and metabolism, including abnormalities of cytosolic processes (i.e., glycolysis and the pentose phosphate pathway), as well as abnormal mitochondria-dependent processes (TCA cycle and OXPHOS) have been identified in the AD brain [24] in the early phase of the disease. This phenomenon, i.e., aerobic glycolysis, first described in cancer, where even under aerobic conditions, tumor cells shift their energy capacity towards less efficient glycolysis, rather than OXPHOS, likely confers a “competitive” advantage to cells. Growing evidence suggests that this phenomenon may also occur in other cellular contexts, such as AD [51]. Therefore, the fully functional mitochondria of neurons that have survived because they are resistant to Aβ can elicit an adaptive response to compensate for brain energy deficits observed in subjects suffering from AD.

It follows that the widely observed mitochondrial dysfunction contributes to increased oxidative stress and reduced ATP synthesis, ultimately affecting the anatomy and physiology of neurons, causing their death [52]. Mitochondrial impairment is believed to precede the formation of neuritic plaques and NFT, as well as to contribute substantially to the early stages of AD and the onset of cognitive decline and memory loss [17,53,54].

In line with this, the imbalance between ROS production—considered a typical by-product of mitochondrial ETC [55]—and antioxidant power has been observed in AD brains, cerebrospinal fluid, and blood [35]. It has been proposed that defective mitochondria are less capable of generating ATP, but more capable of producing ROS, significantly contributing to the oxidative imbalance observed in AD [24,56]. The hippocampus, the cerebral cortex, and the entire brain overall are districts strongly exposed to oxidative stress due to their high aerobic oxidative metabolism dependent on mitochondrial energy production. Their vulnerability is further increased by the reduced antioxidant defense systems and stimulated by the high content of polyunsaturated fats, which are particularly susceptible to oxidative alterations [57]. In AD, there is evidence of an increase in the oxidation of lipids, proteins, DNA, and RNA, both at central [28,58,59] and peripheral levels [28,60], correlating ROS overproduction to the disease [61,62]. The role of ROS produced by mitochondria was also confirmed by the evidence showing the capability of the mitochondrially localized antioxidant MitoQ to prevent cognitive decline, Aβ accumulation, astrogliosis, and synaptic loss in a triple transgenic mouse model of AD [63]. MitoQ treatment has been shown to increase lifespan and improve health in *Caenorhabditis elegans*, a transgenic model for AD [64].

Surprisingly, increased oxidative stress was observed in post-mortem human brain tissue, although, as the disease progressed and both amyloid and Tau aggregations expanded, the level of oxidative damage appeared to decrease [28,65]. The correlation between oxidative stress and proteotoxicity is a matter of intense research; specifically, transgenic AD models, post-mortem AD brains, cultured cells, and isolated mitochondria have been used to identify and characterize the interaction of Aβ or its soluble oligomers with ROS. There are several lines of evidence showing that Aβ induces and increases oxidative stress [66,67]. Elevated levels of Aβ are found to be associated with increased levels of oxidized products of lipids, nucleic acids, and proteins within the hippocampus and cortex [68,69]. Aβ protein fragments have also been found to stimulate ROS production [28], and both β-secretase activity and Tau hyperphosphorylation are augmented by ROS action [27,28], suggesting that mitochondrial ROS production may actually exacerbate the accumulation of Aβ and Tau aggregates [5]. An opposing view postulates an antioxidant and protective role of Aβ, whereby the peptide scavenges reactive radicals of lipid oxidation, prevents ROS formation through the sequestration of transition metals, or even blocks mitochondrial ROS production [70]. This has led researchers to suggest that Aβ may have increased expression in AD because it acts as an antioxidant against mitochondria-induced oxidative stress [59,71]. Among the different isoforms of the Aβ protein, Aβ1-40 has been shown to have the greatest antioxidant effect, but other isoforms, including Aβ1-42 itself, have also shown antioxidant properties [72].

Even mitochondrial biogenesis is weakened in AD [73,74]: the leading regulator of mitochondrial biogenesis, peroxisome proliferator activator receptor gamma-coactivator 1α (PGC-1α), is downregulated at the mRNA and protein levels, particularly in human AD hippocampus tissues and in cell models overexpressing the Swedish APP mutation [75].

The brains of AD patients also show altered mitochondrial dynamics with imbalance in fission and fusion processes, especially in the parietal lobe, which shows early and consistent hypometabolism [76]. Hypometabolism in AD, which is also reflected in the reduced expression of mitochondrial and nuclear genes encoding subunits of ETC complexes [9,77], is one of the most consistent and early abnormalities observed in AD, appearing before the onset of memory deficits [35].

Imbalances in mitochondrial fusion and fission proteins are biased towards mitochondrial fission and therefore towards mitophagy [78]. This increase in fission becomes even more pronounced with a pathological increase in Aβ and pTau levels and their interaction with regulators of mitochondrial division during disease progression [79]. In AD, mitochondrial fission is promoted by increased levels of pro-fission proteins dynamin-related protein 1 (Drp1) and mitochondrial fission 1 protein (Fis1) and decreased levels of the pro-fusion proteins optic atrophy type 1 (Opa1), mitofusin 1 and 2 (Mfn1, Mfn2) [80]. Mitochondrial transport and degradation through autophagy (mitophagy) are also found dysregulated in AD [81].

Furthermore, a study on mitochondrial morphology—essential for maintaining OXPHOS—using brain tissue from different AD models, confirmed mitochondrial fragmentation, associated with increased levels of Drp1 and Fis1 and reduced levels of Opa1, Mfn1, and Mfn2 [82]. Each of these dysfunctional processes—which are observed very early in the neurodegenerative condition and often precede the development of both amyloid plaques and NFT (see [28])—leads to synaptic deficits and critical consequences not only for individual neurons but even for a more complex structure such as the brain [83]. Among other things, just a consideration, the extent and critical role of these deficits in initiating mitochondrial dysfunction may show wide variability depending on the particular biological, environmental, and genetic characteristics of each patient with AD. However, each of these defects can cause the others, exacerbating neuronal dysfunction and therefore neurodegeneration (see [24]).

There are various therapeutic approaches in use aimed at curbing mitochondrial dysfunction and oxidative stress. New antioxidants and agents showing a protective action on mitochondria have been developed [84,85,86], as well as genetic therapies targeting mitochondrial DNA mutations and dynamics (for ref, see [87]), nanotechnology-based drug delivery systems for the targeted delivery of antioxidants and mitochondrial protective agents [25,88,89], and stem cell therapy using induced pluripotent stem cells to supply injured or damaged neurons [90,91]. Furthermore, mitochondrial transplantation, an encouraging but still developing technique, is being explored to substitute dysfunctional mitochondria with healthy ones [89,92].

## 3. Aβ in the Cell Interacts with Mitochondria and Causes Complex I Dysfunction

Although extracellular Aβ deposition is the key histopathological hallmark of AD, it is now established that Aβ enters the cell and accumulates in mitochondria via a mitochondrial import mechanism [93,94], where it has been blamed for any mitochondrial impairment, from respiratory chain defects to the morpho-functional and dynamic imbalance of the organelles (for refs, see [24,95]). How could a small peptide be responsible for so much chaos?

Here, we will examine the mitochondrial abnormalities in AD samples caused by Aβ and we will reserve a special and focused attention for the crosstalk between Aβ and mitochondrial complex I, discussing in more detail the recent advances—as well as those that are dated, but little appreciated in the literature—in order to understand the mechanisms that underlie the dysfunction of the ETC in a broad sense.

### 3.1. APP, the Protein That Produces Aβ

The formation and accumulation of Aβ are generally associated with the clinical manifestations of AD, and the Aβ cascade hypothesis is the main pathogenetic model of AD [96,97,98]. Aβ is generated, by sequential action of β- and γ-secretases [99], from the C-terminal end of its precursor, APP, whose disruption in normal function could contribute to the pathogenesis of AD [100]. APP is a relatively large glycoprotein that crosses the cell membrane from which it protrudes with a large N-terminal domain on the external surface and has several interaction domains with other proteins and with metals, such as copper and zinc. The C-terminal portion of APP inside the neuron is much shorter and is also capable of interacting with various intracellular proteins. Studies carried out using animals and in vitro cell cultures have shown that this protein plays numerous activities which change depending on the period of development of the nervous system. APP is not only important for CNS maturation, but also participates in cell contact and adhesion, neuronal morphogenesis, maintenance of synaptic transmission and plasticity, and can also induce a neurotrophic effect and cell growth [101,102,103]. Consequently, APP knockout mice develop behavioral and cognitive impairment and undergo death during development [104,105,106].

APP is the typical example of proteins which, like Dr. Jekyll and Mr. Hyde, are apparently beneficial and ‘generous’, but can transform into harmful and dangerous ones. In fact, the processing of APP, by α-, β-, and γ-secretases, can lead to the production and release into the extracellular medium of a ‘good’ protein, α-APP—this is the non-amyloidogenic pathway [107,108]—and of some non-toxic peptide derivatives whose function is still largely unknown; conversely, it can also generate the ‘bad’ protein, β-APP, accompanied in a rapid proteolytic sequence by the formation of toxic peptides of which Aβ it is the main component [109,110]. In detail, cleavage by the γ-secretase complex—the amyloidogenic pathway—generates multiple Aβ species ranging from 38 amino acids in length up to 42 amino acids [111], of which Aβ42 is considered the species most prone to aggregation. In turn, Aβ42 is cleaved by β-secretase releasing a soluble fragment (sAPPβ) and a carboxy-terminal fragment (CTF), named C99, inside the membrane [112], that is further cleaved by γ-secretase releasing the Aβ peptide and the APP intracellular domain (AICD). Even the non-amyloidogenic pathway involves the proteolysis of APP by α-secretase within the Aβ region; APP processing generates, in addition to sAPPα, a C-terminal fragment 83 (C83) [112]. Further cleavage of C83 by γ-secretase produces the p3 peptide and the AICD fragment [113]. Several data highlight not only Aβ’s neurotoxicity, but also the neurotoxic action of CTFs which can also seriously compromise neuronal survival. In particular, a possible critical pathogenetic role in AD of the AICD protein fragment, generated from C83 or C99 fragments, identified in the brain of transgenic mice overexpressing human APP (hAPP) as well as in AD brain tissue should be evaluated [113,114].

sAPPα is neurotrophic and neuroprotective protein fragment [115], which also plays a protective action against the toxicity of Aβ [115], while instead Aβ aggregates into fibrils that accumulate between one cell and another where they exert a toxic action on the surrounding healthy cells [116,117]. Although it is not yet entirely clear in which form Aβ exerts its toxic action, whether monomeric, dimeric, or polymeric, the scientific community recognizes the excessive production of this peptide as the trigger for the complex series of events that lead to neuronal death. Consistently, it is important to highlight that in vivo reduction of Aβ is associated with improved memory and less cytotoxicity [118,119].

### 3.2. Mitochondria Are Sites of Accumulation of Both APP and Aβ in AD Neurons

Numerous in vitro and in vivo studies documented that APP and Aβ co-localize with mitochondria [70,92,120,121] and cause structural and functional damage to mitochondria [2,122], preventing physiological neuronal activity [2,37,52,121,123,124,125]. However, if APP and Aβ influence mitochondrial function and bioenergetic pathways, the opposite is also true: mitochondrial function and bioenergetics modulate APP processing and trafficking pathways. On the other hand, it is also known that oxidative stress promotes the production of Aβ by stimulating the activity of β-secretase [66,126].

All these experimental works support the usefulness of using antioxidants for the prophylaxis and treatment of the disease; however, there is a lack of ascertained evidence proving their clinical effectiveness [84,127,128,129].

#### 3.2.1. APP Accumulates into Mitochondria

The first evidence of the localization of APP in mitochondria was observed in the outer membrane of mitochondria from the brains of AD patients by immunohistochemical analyses [130]. Later, Manczak et al. [8,78] using immunoblotting, fractionation with digitonin, immunofluorescence, and electron microscopy techniques, found a relationship between mutant APP derivatives and mitochondria in brain slices of Tg2576 mice—a mouse model overexpressing a mutant form of APP (isoform 695) with the Swedish mutation (KM670/671NL), causing amyloid plaques and progressive cognitive deficits—and in mouse neuroblastoma cells expressing mutant human APP. These data are in line with the findings of Crouch et al. [130] and Caspersen et al. [131], according to which Aβ was also localized in mitochondria: both monomeric and oligomeric forms of Aβ were found to be associated with mitochondria in Tg2576 mice and in N2a cells expressing human mutant APP. In in vitro (human cortical neurons or HCN-1A neurons) and in vivo (Tg2576 mice) studies, Anandatheerthavarada and colleagues [132] found a mitochondrial localization of wild-type and mutant APP (with Swedish mutation). Wild-type and/or full-length mutant APP have also been identified in the mitochondria of PC12 cells and HEK293 cells that have been stably transfected with Swedish APP751 and APP695 [31]. APP was found to be associated with mitochondria and mitochondria-associated membranes (MAMs) [94], i.e., contact sites between the endoplasmic reticulum (ER) and mitochondria that regulate mitochondrial function, calcium flux, and mitophagy. Electron microscopy data have also suggested an association of APP with translocases of the outer (TOM40) and inner (TIM23) mitochondrial membrane, suggesting that APP “clogs” the import machinery of nuclear-encoded mitochondrial proteins [92,133].

#### 3.2.2. γ-Secretase Is Found within the Mitochondria

In addition to mitochondrial APP localization, mitochondria also reportedly contain a functional γ-secretase (co-localization of BACE1 with mitochondria in vitro [134]). Mitochondrial γ-secretase cleaves CTF83 and, in the study by Pavlov et al. [135], the possibility that the CTF83 topology of APP generates AICD and Aβ within the mitochondria was considered. This is an important question because Aβ is also found within mitochondria where it interacts with mitochondrial proteins, altering mitochondrial function [121]. The extremely low mitochondrial levels of substrate (APP) and protease (BACE1 and components of the γ-secretase complex) would rule out that the process of generating Aβ from APP occurs within the mitochondria, hypothesizing rather that Aβ, generated in other organelles, could be transported into the mitochondria [136]. However, in a recent study, Pavlov et al. provided evidence that APP is a substrate of mitochondrial γ-secretase in human neuroblastoma SH-SY5Y cells in culture [135]. The γ-secretase complex has been found in MAMs, suggesting that this may be a potential site of Aβ production. Consistently, several studies report a functional γ-secretase complex within the mitochondria capable of generating Aβ (see [121]). However, consistent with the findings of Mamada et al. [122], Del Prete et al. [137] reported that γ-secretase components, although present in MAMs, which are in close contact with mitochondria, are scarce in purified mitochondria.

#### 3.2.3. Aβ Accumulates in the Mitochondria

The physical interaction of Aβ with mitochondrial protein targets requires the presence of Aβ within the mitochondria or in their close proximity. Therefore, to clarify the effect of Aβ on mitochondrial physiology, it is essential to understand how Aβ reaches the mitochondria.

The accumulation of Aβ in the mitochondria from post-mortem AD brains, cell models, and transgenic mice has been well documented, providing evidence that Aβ is physically localized in the mitochondria and interacts with mitochondrial proteins causing their dysfunction [24,121,138,139,140]. However, it is unknown whether Aβ could be translocated into the mitochondria, generated within mitochondria, or both.

**Figure 2:**
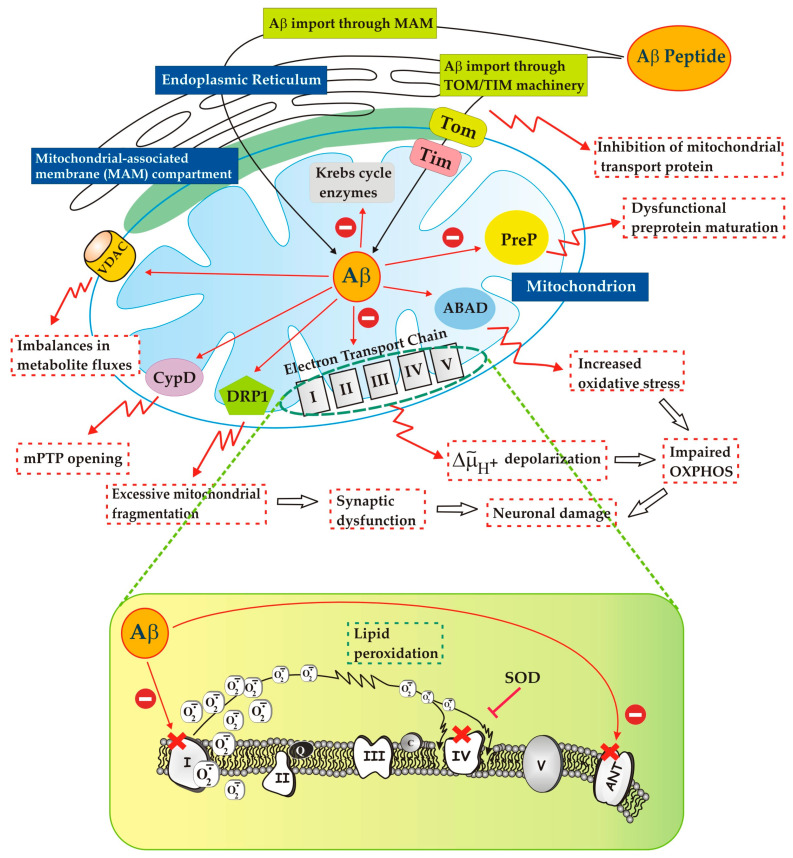
Harmful Interplay between Mitochondria and Aβ in AD. The upper part of the figure graphically describes the path adopted by Aβ to enter the mitochondria through the TOM/TIM machinery or/and crossing the MAM compartment. The central part of the figure shows the multi-targeted mitochondrial proteins inhibited by Aβ, inside the mitochondria. The lower part of the figure gains the schematic view of ETC showing the direct inhibition by Aβ of ANT and complex I, exacerbating ROS production by the complex. The indirect inhibition by Aβ on cytochrome c oxidase (COX) is mediated by ROS-induced damage to mitochondrial lipids (for reference, see [144]).

What is certain is that before accumulation in the mitochondria, Aβ must be accumulated in the cytosol of the cells. In this regard, many studies have shown an interaction of Aβ with various receptors in the cell membrane of the vascular system, neurons, oligodendrocytes, and glial cells where it is transported from the cell surface into the endosomal and lysosomal compartments [45,132] and, increasingly overwhelming evidence reports that the mitochondria are also sites of accumulation of Aβ in AD brains and in the brains of AD mouse models (for ref, see [2]). Interestingly, Anandatheerthavarada et al. [141] hypothesized that APP accumulates within mitochondrial transport channels and undergoes proteolytic cleavage by the Omi protease situated in the mitochondrial intermembrane space. Otherwise, the C-terminal part of APP outside the mitochondria could be cleaved by α/β-secretase, producing an APP fragment immobilized in the outer membrane that would be further processed by γ-secretase, found to partially localize in the mitochondria, resulting in the production of Aβ peptides in the inter membrane space (IMS). However, evidence for intra-mitochondrial Aβ synthesis has yet to be established experimentally. Another possibility is that Aβ could be transported outside of the cell via endocytosis and vesicular transport and released in close proximity to the mitochondria. In this regard, using neuroblastoma cells, it has been demonstrated that extracellular Aβ can be taken up and subsequently localized in the mitochondria [93]. The proposal that Aβ can be translocated directly from the MAMs to the mitochondria through the contact sites between these organelles [142] would also be plausible. Hu et al. [143] propose that the process of mitochondrial Aβ accumulation begins when cytosolic Aβ is recognized by Tom22, then transferred to Tom40 and transported through the TOM channel into the mitochondria, not excluding other possible origins of harmful mitochondrial Aβ (Figure 2).

Other studies have confirmed that Aβ1-40 and Aβ1-42 can be imported in vitro into mitochondria via the TOM complex in a manner similar to a typical mitochondria-targeted protein: both antibodies directed against components Tom20, Tom40, and Tom70 of the TOM complex and those against VDAC and cyclosporin A inhibited Aβ import [94]. This hypothesis was supported by the fact that the helical structure that Aβ adopts in the membrane environment was sufficient for recognition by the TOM complex [145]. Petersen et al. [93] added further information: imported Aβ was mostly present in the cristae and isolated fractions of the inner membrane, but also in the matrix, as confirmed by Walls et al. [145], as Aβ co-localizes with the mitochondrial matrix protein Hsp60 in mouse and human samples. Consistently, using confocal microscopy, it has been shown that Aβ fragments co-localize with complex II of the ETC, supporting the hypothesis that Aβ is able to pass through the mitochondrial membrane [144]. In addition, fractionation studies with digitonin have indicated that Aβ is more abundant in the mitoplasts (inner membrane plus matrix) and less abundant in the outer membrane of mitochondria [8], strongly indicating that Aβ does indeed enter the mitochondria. However, the observation by Pavlov et al. [144] that APP import is arrested due to an acidic domain in amino acids 220–290, leaving the Aβ region outside the membrane, makes it unlikely that Aβ is produced locally in the mitochondria. Added to this is the fact that γ-secretase cleaves its substrates by intramembrane proteolysis; therefore, the localization of APP excludes that it can act as a substrate of γ-secretase in the mitochondria. From this it can be deduced that the Aβ found in AD mitochondria was absorbed [144].

### 3.3. Both APP and Aβ in Mitochondria Alter Their Function

Here, we will focus on the consequences of APP and Aβ peptide accumulation in the mitochondria and their involvement in AD pathogenesis.

Growing evidence suggests that the accumulation of APP and Aβ in synaptic mitochondria from post-mortem AD brains, as well as cellular and transgenic mouse models [146,147,148], due to interactions with proteins essential for proper organelle function [79,83,149,150,151], causes both structural and functional mitochondrial damage, triggers synaptic lesions, interrupting synaptic transmission [152,153], and ultimately prevents neurons from functioning normally [154] (Figure 2). This means that the approach to curb the accumulation of Aβ in the mitochondria may have great pharmaceutical potential.

Before delving into the intricacies of this complex system of interactions, it must be said that if it is true that the accumulation of Aβ in the mitochondria leads to mitochondrial malfunctions, it is equally true that the mitochondrial malfunction, through the generation of ROS, causes an increase in Aβ production, initiating a vicious cycle; in other words, there is a bidirectional relationship between Aβ aggregation and mitochondrial dysfunction. Therefore, pathogenic mitochondrial alterations in AD are likely to be a consequence of a cumulative effect in which mitochondrial dysfunction and Aβ accumulation influence each other [94,155].

In this regard, it has also been proposed that mitochondrial dysfunction and altered metabolism are the first pathogenic alterations observed in AD, a hypothesis which is supported by several studies [30,36,155,156,157]; however, these alterations in metabolism are independent of Aβ and precede the formation of amyloid plaques [24,47,110,119,124,158].

In general terms, both proteins (APP and Aβ) present in the mitochondria trigger mitochondrial dysfunction through a series of pathways, such as by interacting with components of the protein import machinery (translocase of the outer membrane–translocase of the inner membrane (TOM–TIM)) [24,134,159], and blocking the mitochondrial translocation of nuclear-encoded proteins [45,159,160,161,162], like ETC components [93,134,161,162,163], thus compromising mitochondrial functionality. However, they are also capable of disrupting mitochondrial function in other ways, e.g., directly influencing the activities of ETC complexes, such as cytochrome c oxidase (COX) [134,164,165,166], complex I [134,140,148], or the F1α subunit of ATP synthase [5,119], thus compromising mitochondrial respiratory function and ATP production [41,54,57,67].

An inevitable consequence of ETC dysfunction is oxidative stress, which has been observed to increase the accumulation of Aβ in AD [167,168]. In particular, Birnbaum et al. [168] suggested that increased ROS generation at the level of ETC complexes I and III may play a fundamental role in the development of sporadic Alzheimer’s disease, before the onset of Aβ and Tau pathology. Paradoxically, in vivo studies have shown that Aβ can reduce oxidative stress [87].

In his study, Manczak et al. [8] observed that hydrogen peroxide levels were significantly increased in Tg2576 mice, compared to age-matched WT littermates, and directly correlated with soluble Aβ levels in Tg2576 mice, suggesting that soluble Aβ can be responsible for the burst of oxidative stress. COX activity was also reduced in Tg2576 mice suggesting that mutant APP and soluble Aβ impair mitochondrial metabolism in the development and progression of AD. Both events, i.e., increased hydrogen peroxide and decreased COX activity were found in young Tg2576 mice before the appearance of Aβ plaques, suggesting that early therapeutic interventions targeting mitochondria may be effective in delaying the progression of AD in elderly individuals and in the treatment of patients with AD.

In transgenic AD mouse models, two different methods have been developed to reduce ROS production: (1) Tg2576 AD mouse model overexpressing a mitochondrial targeted catalase is a method that marks hydrogen peroxide production [121], and (2) Tg19959 mouse model overexpressing a manganese superoxide dismutase is a method targeting superoxide generation production [2]. Decreases in ROS overproduction led to a reduction in Aβ plaques in transgenic AD mouse models with alterations in the expression levels of secretase enzymes and full-length APP [169]. Overall, these studies suggest that ROS influence APP processing and Aβ production.

Deficits in mitochondrial morphology or dynamics have been observed in fibroblasts from sporadic AD patients [170,171,172,173,174], in mice overexpressing APP [170,175], or even in experimental AD models linked to Aβ peptide treatments. In patients with AD, accumulations of Aβ and the interaction of Aβ with Drp1 are crucial triggering factors leading to mitochondrial fragmentation, aberrant mitochondrial dynamics, and synaptic damage [174]. Consistently, Sheng et al. found that overexpression of APP in neuroblastoma cell lines showed fragmentation and an anomalous distribution of mitochondria caused by an imbalance in the mitochondrial fission and fusion system, leading to mitochondrial and neuronal dysfunction [74]. Reduced levels of Fis1 and increased levels of Drp1, Opa1, Mfn1, and Mfn2 were detected in the hippocampal tissue of AD patients [81]. Specifically, Blagov et al. demonstrated that Aβ—both oligomeric and monomeric forms—interacted with the mitochondrial fission protein, Drp1 [81]. Reddy’s laboratory crossed Drp1 +/− mice with AβPP transgenic mice (Tg2576 line) and created double mutant mice—Drp1 +/− xAβPP in which he revealed, by performing mitochondrial functional assays, that mitochondrial dysfunction is reduced compared to AβPP mice, suggesting that reduced Drp1 protects against Aβ toxicity, mitochondrial dysfunction, and synaptic damage in AD [176].

In addition to Drp1, other targets of mitochondrial Aβ that have been identified and characterized are cyclophilin D (CypD), Aβ-binding alcohol dehydrogenase (ABAD), voltage-dependent anion channel (VDAC), and human Presequence Protease (hPreP) (for refs, see [94]). It has been shown that CypD is able to form complexes with Aβ within the mitochondria of cortical neurons of APP transgenic mice, increasing the translocation of CypD from the matrix to the inner membrane [177,178], an essential process in the opening of mPTP [160], therefore causing dissipation of the internal membrane potential and the generation of ROS, with subsequent rupture of the external membrane and the non-specific release of intermembrane space proteins into the cytosol, which activate various signal transduction pathways such as apoptosis (see [134,178]). Indeed, the deletion of CypD leads to reduced Aβ-induced apoptosis and improves cognitive performance in transgenic mice [179].

ABAD–Aβ complexes were detected in AD brains and in APP/ABAD mutant Tg mice (Tg mAPP/ABAD). Lustbader et al. [180] reported that ABAD directly interacts with Aβ in the mitochondria of AD patients and transgenic mice. ABAD uses NAD^+^ and/or NADH as its cofactor and catalyzes the reversible oxidation and/or reduction of the alcohol group in its substrates [181,182]. The interaction between ABAD and Aβ reduced nicotinamide adenine dinucleotide (NAD^+^) binding and impaired cognition in transgenic AD mice [160].

Therefore, Aβ blocks ABAD activity causing mitochondrial dysfunction and ultimately cell death. Cultured cortical neurons from Tg mAPP/ABAD mice show increased ROS production and decreased mitochondrial membrane potential, ATP levels, and COX activity. The Aβ–ABAD interaction triggers ROS overproduction, cell death, as well as memory and spatial learning defects in 5-month-old APP/ABAD double transgenic mice [183]. Consistently, Lustbader et al. observed that oxidative stress was ameliorated by blocking the interaction between ABAD and Aβ [180]. Inhibition of the interaction between ABAD and Aβ improves mitochondrial function and reduces Aβ accumulation [138].

The interaction of Aβ with VDAC1 demonstrated by Manczak and Reddy [183] as well as by Smilansky et al. [184] determines the detachment of hexokinase, an anti-apoptotic protein, an increase in channel conductance, probably inducing the oligomerization of VDAC1, and the release of cytochrome c (for refs, see [185,186,187]). However, it has also been shown that the interaction of VDAC1 with Aβ can lead to channel closure. This occurs because VDAC1 also interacts with phosphorylated Tau, another key component in the pathogenesis of AD, which, together with Aβ, leads to channel blockade [184]. This Tau–Aβ–VDAC interaction leads to imbalances in metabolite fluxes through the outer mitochondrial membrane and consequently to defective OXPHOS. Whether Aβ increases or decreases VDAC1 conductance appears to depend on the staging of the disease, i.e., whether phosphorylated Tau appears or not [185]. However, the role of VDAC1 dysfunction in the etiology of AD is certain. Downregulation of VDAC1 and/or prevention of its interaction with Aβ and phosphorylated Tau could potentially preserve mitochondrial function, slow AD progression, and ultimately improve cognitive function in AD patients [184,188].

An interesting aspect in which the Aβ peptide appears to be involved concerns the presequence processing of several mitochondrial proteins encoded in the nucleus. Most mitochondrial proteins possess N-terminal presequences required for targeting and import into the organelle which, upon import, are cleaved by matrix processing peptidases and subsequently degraded. Aβ appears to inhibit the degradation of presequence peptides by the mitochondrial peptidasome (PreP) [189]. PreP is a protease localized in the mitochondrial matrix responsible for the degradation of the presequences of imported proteins [189,190,191]: this process of removal of the presequence causes a dysfunctional preprotein maturation and obviously modifies the protein profile of the mitochondria, causing multiple functional anomalies in the organelle in AD. Presequence processing is indeed impaired in the brain mitochondria of AD patients [192], where mitochondrial Aβ is abundant, suggesting pathophysiological relevance to the human disease. Furthermore, considering that human PreP can also degrade Aβ peptides located in mitochondria [190,193], reducing their toxic effects on mitochondria, it follows that the enzyme that degrades Aβ in mitochondria is also a target of the toxic effect of mitochondrial Aβ, thus it is clearly revealed as an important regulator of Aβ concentration within mitochondria: perturbation of its activity can potentially influence Aβ accumulation [194].

It is known that Aβ-mediated mitochondrial dysfunction can also result in impaired calcium homeostasis [37,45], as well the fact that Aβ interacts with mitochondrial matrix components such as Krebs cycle enzymes [195,196,197]. 

In addition to biochemical changes, the mitochondria of AD patients exhibit structural changes, e.g., fragmented mitochondria with abnormal cristae (see [28,67]), or even disruption of mitochondrial membranes and cristae with decreased ATP production [72,198,199]. Furthermore, in relation to the accumulation of Aβ within the mitochondria, a reduction in the quantity and volume of mitochondria was found in mouse hippocampal neurons treated with Aβ [34]. Loss of mitochondrial mass has also been measured in brain lysates and hippocampal regions of AD patients [78,200]. However, it should be kept in mind that in the hippocampus, the reduction in mitochondrial mass could be a consequence of other dangerous events triggering the disease or undetectable but still toxic levels of Aβ, since the reduction in mass has already been found in 3-month-old mice, when there was not yet amyloid overload. Reduction in the mitochondrial mass could be a consequence of a diminished mitochondrial biogenesis rate [78] or it could be due to increased mitochondrial degradation [78]. Even in the APP/PS1 mouse model, the mitochondrial mass was significantly decreased compared to WT mice. This decrease was detected in the hippocampus of 3-month-old mice and subsequently also in the cerebral cortex, indicating that the reduction in mitochondrial mass due to Aβ overload can be considered an early event in the development of AD.

### 3.4. Aβ Interacts with Complexes I and IV of the Mitochondrial Respiratory Chain

The mitochondrial respiratory chain consists of five enzymatic complexes localized in the inner membrane of the mitochondria. Reduced equivalents of NADH and FADH_2_ derived from the oxidative metabolism of carbohydrates and fatty acids flow from complex I and II, respectively, to complex IV through a series of redox reactions. The energy released during this process, in the form of an electrochemical proton gradient, directs the FoF1ATP synthase (indicated as complex V) to produce ATP from ADP and inorganic phosphate. Since the 1990s, it has been demonstrated, using post-mortem brain tissues, as well as platelets from AD patients and AD cybrid cells, that the ETC function is reduced in AD [201,202,203]. The discovery of the reduced toxicity of Aβ in cells depleted of mitochondria (rho-0) supports the hypothesis that the toxicity of the peptide is mediated by the ETC [204].

The decrease in activity was found in each ETC complex, although the most significant impairment occurred in multiple areas of the AD brain at the level of the complexes I—the largest one of the mitochondrial respiratory chain—and IV, i.e., COX [77,205,206,207,208,209,210]. In contrast to complexes I and IV, the evidence for deficiencies in complexes II, III, or V activities in AD was not as pronounced. However, a decrease in complex II activity was reported in the APP/PS1 mouse model [211], and protein levels representative of all five ETC complexes were decreased in the piriform and insular cortical regions of 3xTg mice, before the onset of detectable plaques [212]. Slowing of ETC leads to ROS formation at complex I [25,44,213], thus linking ETC inhibition and ROS accumulation in AD and suggesting that mitochondrial complex I dysfunction may contribute to the pathogenesis of sporadic AD.

However, findings on how ETC complexes are affected by Aβ are rather inconsistent because they suggest different sites along the ETC as the site of dysfunction, with some studies reporting complex IV dysfunction [214,215], while others report that complex I (NADH dehydrogenase) is the site most implicated in Aβ dysfunction [25,165,215,216].

In neuroblastoma cells, overexpression of APP leads to elevated levels of Aβ1-40 with reductions in cellular respiration, ATP levels, and COX activity; the activity of complex III was also high [217]. In PC12 cells, exogenous Aβ depolarizes the mitochondrial membrane potential and decreases the activities of ETC complexes I, III, and IV, also reducing oxygen consumption [218]. In primary cortical neurons, shorter fragments of Aβ, e.g., Aβ25-35, reduce cellular ATP production, antioxidant levels (glutathione, GSH), mitochondrial membrane potential, and the activities of ETC complexes [219] (see Table 1). Conversely, in isolated rat brain mitochondria, the same short peptide Aβ25-35 reduced COX activity but not complex I, II, or III activities (see [169]).

In AD transgenic J20 mice, Caspersen et al. [131] observed that mitochondrial Aβ was associated with complex III and IV dysfunction. In 3-month-old AβPP transgenic mice, decreased complex IV activity was observed in the absence of plaques, but accelerated substantially with increasing age, as did Aβ plaque burden [220]. The decreased activity of complex IV, also observed in the post-mortem brain of AD patients (see [39]), is in line with the evidence showing that Aβ, mainly monomeric and oligomeric forms, interacts with subunit 1 of this complex [165], probably resulting in its reduced activity. Furthermore, studies conducted in the AβPP/PS2 transgenic mouse reveal a reduction in complex IV activity, while in the pR5 mouse (a Tau transgenic mouse), the reduction was observed in complex I [222]. Mitochondrial respiration and bioenergetics were more severely impaired by crossing AβPP/PS2 and pR5, thus showing a synergistic effect between Tau and Aβ in AD [222]. Further in vivo studies found that genetic knockdown of COX10 in AD transgenic mice reduced Aβ plaque burden [223]. Interestingly, COX Vmax and function were reduced in AD subjects both cerebrally and systemically [163,205,224,225], suggesting a strong relationship between COX function and Aβ production.

In cells in which the respiratory chain is missing due to the absence of mitochondrial DNA (mtDNA), i.e., ρ0 cells, no ROS production, caspase activation, or cytochrome c release was observed in the presence of Aβ25-35 [226], validating the hypothesis that the harmful effects of Aβ on the mitochondria occur through direct or even indirect interactions with the respiratory chain. 

The studies conducted on the compound tricyclic pyrone (CP2), a small molecule that penetrates the blood–brain barrier, which accumulates in the mitochondria and selectively and specifically inhibits complex I, are very interesting [227,228]. CP2 attenuates Aβ-induced toxicity in primary cortical neurons [229] and reduces Aβ aggregation in a 5× FAD mice transgenic animal model [230]. Inhibition of complex I by CP2 reduces both Aβ and pTau levels and prevents the development of the cognitive and behavioral phenotype in three mouse models of AD [228], suggesting that modulation of complex I activity represents a therapeutic strategy promising for AD. In this regard, CP2 has been shown to alleviate the cognitive and pathological deficits in animal models of AD, indicating the potential use of complex I modulators in the treatment of AD [231,232]. Generally, across all treatment paradigms, CP2 improved energy homeostasis in the brain and periphery, synaptic activity, dendritic spine maturation, cognitive function, and proteostasis (reduction of Aβ and pTau levels), by interfering with the formation of Aβ aggregates [229,230,233], as well as reduced oxidative stress and inflammation in the brain and periphery, ultimately blocking neurodegeneration [227,234]. Furthermore, increased ATP levels together with reduced ceramide levels were consistent with improved brain energy homeostasis in AD patients [227]. In neurons from PS1 and APP/PS1 mice, CP2 treatment increased mitochondrial dynamics and function, including the restoration of axonal trafficking [228].

Overall, these studies support the hypothesis that the partial reduction of the activity of complexes involved in OXPHOS and the ETC mechanism, using genetic or pharmacological down modulation approaches, provides significant health benefits, improving mitochondrial function and cellular energetics in multiple in vitro and in vivo model systems.

In this regard, it is interesting to underline that inhibition of mitochondrial respiration has in many cases been found to increase lifespan and protect against tissue damage, a phenomenon probably attributable to the decrease in the mitochondrial production of reactive species (see [235]). Since this strategy has also been shown to improve health and lifespan, the development of safe and effective complex I inhibitors could promote healthy aging by delaying the onset of age-related neurodegenerative diseases. These data suggest that it is possible to develop safe and effective complex I inhibitors that are target-selective and do not induce mitochondrial dysfunction associated with increased ROS production. In this regard, more than 60 complex I inhibitors have shown a differential effect on the enzymatic kinetics or on the production of ROS, so much so that molecules including rotenone, piericidin A, and rolliniastatin 1 and 2 increase ROS, while others, such as stigmatellin, mucidin, capsaicin, and coenzyme Q2 prevent its formation [236]. Similarly, there are mutations in complex I that preserve the conversion of NADH to NAD^+^—the ratio of NADH to NAD^+^ determines the rate of superoxide formation—and, therefore, the activity of complex I by completely blocking the pathological production of ROS [237].

In a recent study, Olajide et al. [221] evaluated the exposure to 1 μM hAβ1-42, for a period of 3 h, on the functions of respiratory mitochondria and the expression of key mitochondrial and synaptic proteins in the EC, considered among the first cortical regions to be affected by AD pathology [221,238,239]. Wild-type EC slices revealed a marked reduction in oxygen consumption and, in particular, complex-I-related activity was markedly reduced by hAβ1-42 (see Table 1). Furthermore, hAβ1-42 reduced the immunoexpression of both mitochondrial superoxide dismutase (SOD2)—this is consistent with a rapid increase in mitochondrial ROS production induced by hAβ1-42—and cytochrome c, ultimately culminating in oxidative stress and synaptic dysfunction [17,41,240,241].

That complex-I-specific dysfunction is implicated in AD is also highlighted by the fact that incomplete fragments of complex I have been found in AD brain samples (for refs, see [25]), likely due to the incorrect assembly of complex I. An international team of scientists led by the European Synchrotron Radiation Facility [242] joined forces with scientists from the Institut de Biologie Structurale (CNRS, CEA, Université Grenoble Alpes), the Grenoble Institut des Neurosciences, and the European Laboratory of Molecular Biology (EMBL), to study the proteins involved in respiratory complex I which, being the first enzyme of the respiratory chain, is the main source and target of ROS and deficiencies in activity, often characterized precisely by defects in the complex I assembly process [243], leading to the most common OXPHOS disorders in humans [244]. The scientists focused on a protein called ECSIT, which plays a critical role in the immune system and also appears to establish interactions with many proteins connected to mitochondrial bioenergetic activity widely compromised in AD, finding that it has an important role in assembling the complex ‘helper’ which will assemble the respiratory complex I. ECSIT, as a ‘site manager’, directs the function of the proteins that are part of the ‘helper’ complex so that they do the job they are supposed to do well. One of these proteins is ACAD9, a protein that can oxidize fatty acids, acting as an acyl-CoA dehydrogenase enzyme in the first step of the fatty acid β-oxidation pathway, or it can assemble the respiratory complex [242]. Soler-López and her coworkers found that ECSIT turns off ACAD’s oxidative function, so the protein could exclusively direct its activity to assembling the respiratory complex [244]. If ECSIT did not exert any action, it would be chaos, as proteins would be doing different things at the same time; therefore, ECSIT plays a key control role in the whole respiratory complex, and as a result, in mitochondrial activity. Furthermore, noteworthy first clues reveal that ECSIT has been identified as a molecular node that interacts with Aβ-producing enzymes [245], implicating a potential role in the pathogenesis of AD. This association of ECSIT with Aβ-producing enzymes suggests that a reprogramming of mitochondrial bioenergetics may be implicated in the early stages of AD, but this is still being investigated.

Going back in time, the study by Bobba et al. [139], conducted 10 years ago, in addition to confirming that Aβ inhibits ETC, has the added dimension of attempting to shed light on the mechanism underlying Aβ-induced mitochondrial ETC impairment, given that up to that point, the work of various groups had tried to clarify the molecular mechanisms underlying ETC defects, but without convincing results, precisely because they were conflicting. Understanding the mechanisms of Aβ-induced impairment of mitochondrial complexes I and IV—the complexes most suspected in AD—provides information that will be useful for the development of mitochondria-targeted therapeutic approaches for the treatment of AD. We willingly propose it in this context because—as often happens in the world of scientific research—sometimes the discoveries made, which turn out to be ‘novelties’ when they come to light, are at first glance overlooked—and therefore neglected—perhaps because they were not validated by other studies, only to be re-evaluated/revisited after a long time, because, like pieces of a mosaic, over time they represent the right missing piece and are strictly necessary to reconstruct and validate the mechanism underlying the pathology.

Bobba et al. [139] used a very simple experimental system—perhaps, even in some respects, rudimentary—where the homogenate of cerebellar granule cells (CGCs) were exposed to low concentrations of fibrillar Aβ1-42 in the micromolar range 0.5–2 (for refs, see [246,247]), so as to offer direct information on mitochondrial protein targets, without the use of single, double and, recently, triple transgenic AD mice. The reasonableness of the choice of CGCs as an experimental model is based on the fact that during the onset of the apoptosis of CGCs, several molecular events evocative of AD are induced, such as the activation of the amyloidogenic process, the splitting of Tau with the production of toxic fragments (see [248] and references therein), as well as mitochondrial dysfunction. If the afferents, which normally reach the cerebellar neurons called ‘granules’, are interrupted due to a traumatic rupture of the afferent fibers, a massive apoptosis program is started in them. Or even, if, in vitro, the potassium concentrations, normally used to keep the nerve cells alive, are reduced—the presence of this cation simulates a condition of electrical stimulation—an equal apoptotic process is activated, accompanied by increased production and release of Aβ and extracellular fibril formation. These data obtained in in vitro cultures or in animal models unequivocally confirm that a causal relationship could exist between events activating apoptosis and AD (see above). Furthermore, the literature supports cerebellar involvement in AD and definitively opposes the common practice of using the cerebellum as a negative control in biochemical studies (see [249] and references therein). However, in order to further demonstrate that the experimental approach used represents a very valuable tool to study how Aβ induces the impairment of specific mitochondrial enzymes in a manner very close to what happens in vivo, the authors also measured the activities of ETC complexes in homogenates from post-mortem brains of AD patients.

The finding by Bobba et al. consists in the fact that Aβ selectively reduces both respiration and the ΔΨ generation induced by respiratory substrates of both complexes I and IV [139]. As expected, the reduction in complex I was associated with an increase in intracellular ROS. From the obtained results, two important aspects were highlighted by Bobba et al.: (i) Aβ-induced ROS production is strongly inhibited by rotenone; (ii) complex I is almost exclusively responsible for the production of intracellular superoxide. The fact that SOD—known to “capture” superoxide, thus lowering the level of ROS—almost completely prevented Aβ-induced COX inhibition, proposing that the effect of Aβ on COX was actually mediated by ROS, likely through peroxidation of cardiolipin [164] on which COX activity is strictly dependent [246,250]. Confirming this, COX activity was inhibited by the artificial ROS production system, (i.e., the system Xanthine, XX, plus Xanthine oxidase, XOD) in a manner prevented by SOD [139]. Otherwise, the system (XX + XOD) had no effects on the activity of purified COX, which did not appear to be compromised even by Aβ. These findings support the hypothesis that Aβ, rather than interacting with the active site of complex IV, probably acts on the membrane microenvironment—which obviously does not support the activity of purified COX—causing its lipid peroxidation. Therefore, the steps concerning Aβ in compromising mitochondrial function are: (i) Aβ directly inhibits complex I; (ii) Aβ exacerbates ROS production at the level of complex I; (iii) Aβ inhibits COX through ROS-induced damage to mitochondrial lipids (Figure 2).

However, the possibility that there could also be a direct interaction of Aβ with sites of the COX molecule other than the active one was not excluded. Likewise, it was not excluded that complex I itself could be influenced by the ROS of its own production, thus starting a vicious circle. 

Another particular aspect of the study by Bobba et al. [139] concerned the direct interaction of Aβ with complex I. At that time, a case of direct interaction had already been identified represented by the interaction of Aβ with a Tau fragment (the NH_2_-26-44 fragment), derived from neurotoxic NH_2_ of the human Tau40 isoform (441 amino acids), in human AD synapses, in association with the mitochondrial ANT-1 [247,251]. The two peptides—i.e., Aβ and the NH_2_-26-44 Tau peptide—individually inhibit ANT-1, while together they further aggravate mitochondrial dysfunction by exacerbating ANT-1 impairment and thus causing dysfunctions of energy metabolism [247,251]. However, regarding the interaction between Aβ and complex I, co-immunoprecipitation analysis with a specific antibody directed against the NDUFS3 subunit belonging to the catalytic core of complex I [252] performed on synaptic-enriched fractions—which contain the complete presynaptic terminal, including mitochondria and synaptic vesicles, along with the postsynaptic membrane and postsynaptic density—and using the 4G8 Aβ antibody as bait, revealed no interaction between Aβ and the NDUFS3 subunits of complex I. This does not mean that it is not necessary to hypothesize that the interaction exists in any case, but is labile or transitory, or rather that other specific subunits of this respiratory complex may be involved in the interaction, thus explaining its reduced activity.

## 4. Why This Review? Some Concluding Remarks

Almost daily, growing experimental evidence and original scientific articles bring to the forefront the identification of new key mechanisms or novel molecules, rather than other genetic mutations, linked or not to mitochondrial dysfunction, inflammatory processes, immune dysregulation, or even viral infections and environmental factors, as possible culprits of AD. However, we are all different individuals and even neurodegenerative diseases, specifically AD, can be triggered by different stimuli. Furthermore, the treatment that seems to be appropriate for one person may not be suitable and effective for another; therefore, the therapy will need to be personalized. At present, it is only worth acknowledging the fact that, for years, the biomedical field has struggled to develop new therapeutic choices for AD without significant progress. There is presently no cure for AD and treatment is limited to symptom management [253,254]. However, it is also true that one of the reasons why we do not yet have a cure for this disease is due to the complexity of the human brain, in addition to the complexity of the disease itself.

In this review, we aim to address and discuss the critical role of mitochondrial dysfunction in AD particularly focusing on the aberrant interaction of the mitochondrial complex I with Aβ (Table 1). Targeting mitochondria through pharmacological modulation that reactivates their impaired function may have curative potential for the therapeutic management of the disease. This suggests that mitochondrial dysfunction should be considered a *chance*, rather than a problem, in the complex context of the disease, so as to advance the hypothesis that the cure for Alzheimer’s may lie in the mitochondria.

It is known that neurogenesis—i.e., the formation of new neurons—is a process that reaches its completion in the early stages of neonatal life; therefore, neurons, with the exception of particular neurogenic brain niches in which neurogenesis can occur in adult-hood [255], cannot regenerate and renew themselves throughout life. Conversely, the mitochondria contained in neurons, like those present in all other cell types, undergo constant turnover and renewal. “Neuronal health” is closely linked to mitochondrial function, so as long as the mitochondria remain healthy and functionally efficient, they ensure that neurons can function properly.

Despite the growing evidence showing mitochondrial loss in the neurons of AD patients, the idea that mitochondrial dysfunction may be a key event in AD etiology has long remained marginal and poorly taken into consideration in the research field. More than one reason can explain why there has been this lack of scientific attention on this topic.

One factor that surely played an important role was the fact that a large amount of AD research funding went to scientists studying Aβ and Tau, in order to test the hypothesis that confiscating or reducing Aβ and Tau protein aggregates in the brain could have a strong impact on cognitive function. Added to this was the inadequacy of the methods used to study mitochondria in humans, thus making it difficult to detect, prevent, or treat mitochondrial dysfunction. In more recent years, the idea of AD as a multifactorial disease has gained ground; therefore, thanks to advancing structural biology techniques, a reconsideration of the mitochondria as a valid therapeutic target has been strongly recommended, considering that their functional alterations are one of the factors that can actively contribute to the onset and progression of AD [256]. However, what is still missing is the understanding of the time course and causal sequence of the events that lead to AD, as well as a valid and effective therapy. In recent decades, novel innovative in vitro studies allowed the transfer of mitochondria from healthy cells to damaged cells to rescue those with impaired mitochondrial metabolism in a process that could be applied as a new therapeutic option to repair brain cells affected by AD. So far, the transfer of isolated human mitochondria has been tested on AD animal models, showing that it improves cognitive deficit, neuronal loss, and gliosis, with the concomitant increase in the activities of brain citrate synthase and COX, highlighting an improvement in mitochondrial dysfunction in its entirety [257]. We are far from a clinical trial and a cure, but the prospect of transplanting healthy mitochondria, exactly as if they were spare parts to replace those now compromised by the disease, is very interesting. Therefore, if the neurons affected by AD can be strengthened with young mitochondria capable of producing sufficient energy, the cells could stop accumulating amyloid plaques, blocking the progression of the disease or even eradicating it. All this must be considered within an approach that must be multifactorial.

We hope that the wind of scientific investigation can change, steering the sails of AD research towards directions that are still little explored.

## Figures and Tables

**Table 1:** Relationship between β-amyloid and mitochondrial complex I in different experimental models.

Effect of Aβ on Mitochondrial Complex I	Experimental Model	Reference
Direct inhibitory effect of monomeric and oligomeric forms of extracellular Aβ on complex I Interaction with subunit 1 of the complex	Human neuroblastoma cells	[165]
Deficits in the enzymatic activity of complex I	Hemizygous (+/−)TgMcGill-R-Thy1-APP rats	[215]
Decreased complex I activity	Triple-tg AD mice	[216]
Deregulation of subunits of complex I associated with reduction in the mitochondrial membrane potential	Triple-tg AD mice	[220]
Inhibition of complex I by Aβ_25-35_ with reduction in cellular ATP production and mitochondrial membrane potential	Primary cortical neurons	[219]
Inhibition of complex I by Aβ_25-35_ associated with reduction in mitochondrial membrane potential and oxygen consumption	PC12 cells	[218]
Reduction in complex I activity by hAβ_1-42_ associated with reduction in oxygen consumption and increase in mitochondrial ROS production	Entorhinal cortex	[221]
Reduction in complex I by fibrillar Aβ_1-42_ associated with increased intracellular ROS	Cerebellar Granule cells	[139]

## Data Availability

Not applicable.
